# Experience With Esthetic Reconstruction of Complex Facial Soft Tissue Trauma: Application of the Pulsed Dye Laser

**DOI:** 10.5812/traumamon.16220

**Published:** 2014-08-01

**Authors:** Ali Ebrahimi, Hossein Mohammad Kazemi, Nasrin Nejadsarvari

**Affiliations:** 1 Trauma Research Center, Baqiyatallah University of Medical Sciences, Tehran, IR Iran; 2 Department of Medical Ethics, Tehran University of Medical Sciences, Tehran, IR Iran

**Keywords:** Face, Wounds and Injuries, Soft Tissue Injuries, Esthetics

## Abstract

**Background:**

Facial soft tissue injury can be one of the most challenging cases presenting to the plastic surgeon. The life quality and self-esteem of the patients with facial injury may be compromised temporarily or permanently. Immediate reconstruction of most defects leads to better restoration of form and function as well as early rehabilitation.

**Objectives:**

The aim of this study was to present our experience in management of facial soft tissue injuries from different causes.

**Patients and Methods:**

We prospectively studied patients treated by plastic surgeons from 2010 to 2012 suffering from different types of blunt or sharp (penetrating) facial soft tissue injuries to the different areas of the face. All soft tissue injuries were treated primarily. Photography from all patients before, during, and after surgical reconstruction was performed and the results were collected. We used early pulsed dye laser (PDL) post-operatively.

**Results:**

In our study, 63 patients including 18 (28.5%) women and 45 (71.5%) men aged 8-70 years (mean 47 years) underwent facial reconstruction due to soft tissue trauma in different parts of the face. Sharp wounds were seen in 15 (23%) patients and blunt trauma lacerations were seen in 52 (77%) patients. Overall, 65% of facial injuries were repaired primary and the remainder were reconstructed with local flaps or skin graft from adjacent tissues. Postoperative PDL therapy done two weeks following surgery for all scars yielded good results in our cases.

**Conclusions:**

Analysis of the injury including location, size, and depth of penetration as well as presence of associated injuries can aid in the formulation of a proper surgical plan. We recommend PDL in the early post operation period (two weeks) after suture removal for better aesthetic results.

## 1. Background

Facial soft tissue injuries are amongst the most challenging problems facing the plastic surgeon. The life and self-esteem of patients with facial injuries may be compromised temporarily or permanently. A patient arriving in the emergency room with severe soft tissue facial trauma requires a surgeon with appropriate training in the management of these injuries.

Trauma can lead to complex facial injuries with tissue loss. Motor vehicle crashes make up the main proportion of these injuries; bite wounds and ballistic injuries are the next most common causes of facial injuries. Most defects can be reconstructed immediately leading to better restoration of form and function with early rehabilitation (1-13). Commonly there is a wide range of options for repairing a given defect. These include healing by secondary intention, primary closure, placement of a skin graft, mobilization of local or regional flaps, and free flaps. Local flaps often produce superior functional and esthetic results. A great advantage of local flaps is that the tissue compares closely to the missing skin in color and texture. These flaps can be rotated, advanced, transposed, or interpolated into the tissue defects (14-18).

## 2. Objectives

The aim of this study was to present management of facial soft tissue injuries in different facial zones and use of early PDL.

## 3. Patients and Methods

**Figure 1.**
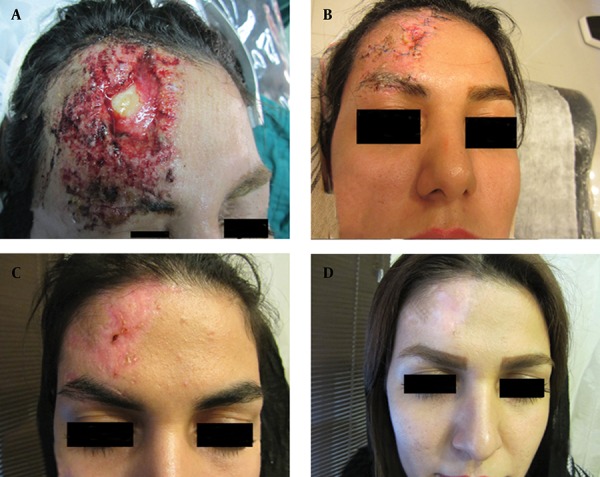
A 30-Year-Old Woman With Severe Forehead Soft Tissue Defect Due to Blunt Trauma. A) Before operation, B) early post operation, C) one month post operation, D) one year post operation.

We studied patients treated from 2010 to 2012 for blunt or sharp (penetrating) facial soft tissue injuries to the different areas of face. We included patients with only soft tissue injuries and those with combined injuries were excluded from this study. All soft tissue injuries were treated with primary intention following debridement, irrigation, and primary surgical intervention by different methods depending on the defect size and location. We explained probable complications of primary intention to all patients and informed consents were obtained from all patients before surgical interventions. The medical ethics committee of our hospital approved the study protocol. We used primary closure, skin graft, and different local flap techniques to fit the location of the injury and compensate for the extent of tissue loss. After 48 h, dressings were removed and all patients received prophylactic oral antibiotics for 24-48 h (a dose before surgery and 48 h after surgery). We had complex soft tissue damage in some of the cases (Figure 1). Early post-operative pulsed dye laser (PDL) was used two weeks post operation in three sessions. The aim of PDL therapy was better esthetic results in all facial suture lines, except in periorbital and ear regions.

Photography from all patients before, during, and after surgery was done. The frequency of the facial soft tissue injuries with regard to the anatomical regions are shown in Table 1. We considered some reconstructive rules for reconstruction of different part of facial soft tissues: 

**Table 1.** The Anatomic Regions of 63 Facial Soft Tissue Trauma Treated From 2010 to 2012 ^a^

Facial Region	Male	Female	Total Number
**Forehead**	5 (7.9)	3 (4.7)	8
**Ear**	5 (7.9)	2 (3.17)	7
**Eyelid and eyebrow**	10 (15.8)	5 (7.9)	15
**Nose**	8 (12.6)	3 (4.7)	11
**Cheek**	7 (11.1)	2 (3.17)	9
**Lips**	6 (9.5)	2 (3.17)	8
**Chin**	4 (6.3)	1 (1.58)	5

^a^ Data are presented as No. (%).

### 3.1. Forehead

**Figure 2.**
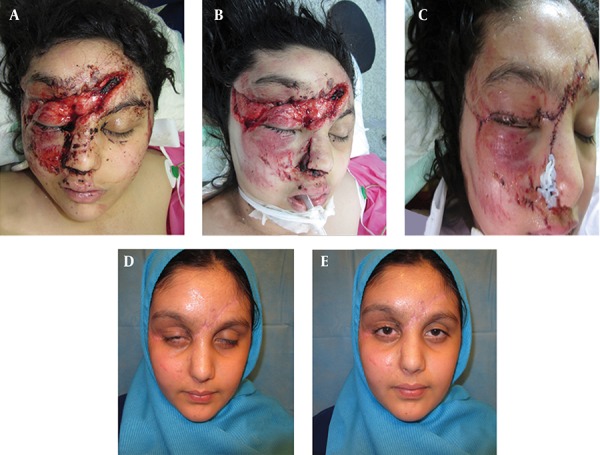
A 10 Year-Old Girl With Severe Complex Forehead, Eyebrow, Eyelid, Nose and Frontal Branch of Facial Nerve Damage After Car Accident. A) Before operation, B) intra operative view, C) early post operation, D&E) one year post operation.

For the repair of soft tissue injuries of the forehead, conservative debridement and washing should be performed in the early stages and the line of repair should be parallel to Langers lines (Figure 1). For the repair, adjacent soft tissue is in priority and should be released and used for esthetic reconstruction. The earlier these injuries are repaired, the better will be the cosmetic results. In some cases with tissue defects, local flap or skin graft was necessary for reconstruction (Figure 2).

### 3.2. Eyebrow

In eyebrow injuries, establishment of anatomical alignment is important. Whereas the medial brow is more important esthetically than the lateral brow, lateral sides of the eyebrow should be used for the reconstruction of medial sides and local flaps should be designed on this basis. There is no need for eyebrow shaving before operation (Figure 2).

### 3.3. Eyelid

The lids have several layers and are very important for globe preservation; therefore, accuracy is required for reconstruction and each anatomic layer must be reconstructed (Figure 2). The appropriate method is selected according to the defect size, location, and the elasticity of the adjacent tissues. The selected technique must recreate the normal eyelid function with good esthetics (19).

### 3.4. Lips

Primary reconstruction is usually possible in lip lacerations. Defects up to 30% can be repaired primarily; however, larger defects require local flaps for reconstruction that is taken from the opposite lip or cheek. The type of flap depends on the location and size of the defect. The first suture should be done in the vermilion and the exact alignment of the lips should be established. Reconstruction of all layers is necessary for a functional and esthetic lip.

### 3.5. Nose

If there is no skin defect in the nasal laceration, primary repair is done. Anatomic alignment should be established exactly. Local flaps or full-thickness skin grafts are used to reconstruct skin defects. The results of local flaps are better than grafts. If the ala and tip are involved, they must be repaired first to prevent notching.

### 3.6. Ear

Ear laceration must be repaired anatomically; we must realign the helical rim initially. If there is a soft tissue defect, local flap reconstruction is necessary. In ear amputation, replantation or staged reconstruction of the auricle must be done.

### 3.7. Facial Skin

**Figure 3.**
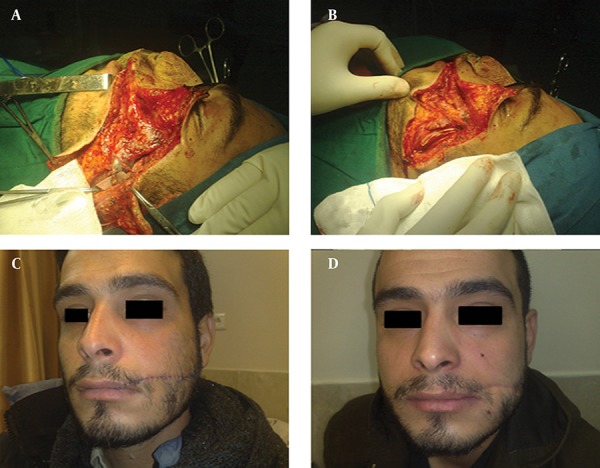
A 25 Year-Old Man With Complex Cheek, Lip, Facial Nerve, and Parotid Duct Disruption Due to a Large Sharp Wound of the Face. A&B) Pre and intra operative view, C) early post-operation after microscopic repair of facial nerve branches parotid duct and laser therapy, D) six months post-operation.

In facial skin injuries, it is important to carefully examine facial nerve branches and the parotid gland duct in initial evaluation; if damaged, it must be repaired with the aid of loop magnification. Exact examination is necessary preoperatively (Figure 3) and facial nerve branches should be carefully repaired. In addition, muscles and subcutaneous tissue must be repaired anatomically with absorbable sutures to prevent a trap-door deformity. Skin Sutures should be removed in the early post-operative period in all repaired areas of the face to leave minimal scars.

## 4. Results

Overall, 63 patients including 18 (28.5%) women and 45 (71.5%) men with the age of 8-70 years (mean age of 47 years) underwent facial reconstruction due to soft tissue trauma in different parts of the face (Table 1). Sharp wounds were seen in 15 (23%) patients and blunt trauma lacerations were seen in 52 (77%) patients. In both sexes, the most common site of injury was the periorbital area and the least common site was the chin. Sixty-five percent of the facial injuries were repaired primarily and the remainder were reconstructed with local flaps or skin grafts from adjacent tissues. We had no post-operative infection, hematoma, ischemia, or necrosis in our patients and the patients were satisfied after the operation. The facial lacerations were managed in the early 24-48 hours after injury. To achieve better esthetic results, all patients underwent PDL therapy two-week post-operation after suture removal in three sessions and followed 6-18 months with no major complications. The esthetic results of the patients were evaluated with clinical examination and photography post-operatively. The results of reconstruction in different parts of the face were good. 

## 5. Discussion

The reconstruction of the skin and soft tissue defects of the face can be challenging due to the, multiple subunits of the face, and the variety of trauma. Injuries to the face can have minor or devastating consequences. The timing, sequence, and application of appropriate surgical procedures and techniques used for reconstruction and rehabilitation of maxillofacial injuries can affect the outcome and aesthetic result (20).

There are several reconstructive options for facial trauma including primary repair, skin grafts, local different flaps, regional flaps, or distant and free flaps. The choice of reconstructive procedure depends on several factors including size, location, and involvement of deeper structures.

The staged sequence of treatment as described by Behnia and Motamedi (20) dictates the timing of both hard and soft tissue treatment and largely depends on surgical judgment and the general condition of the patient. The selection of the appropriate surgical technique is as important as the timing, since incorrect selection or application of surgical techniques may also promote infection, wound dehiscence, graft rejection, facial deformity, and subsequent revision operations. Complications will also prolong hospital stay and increase postoperative morbidity as well as treatment costs.

When facial soft tissue injuries are treated electively, previous scars should be excised and whenever possible, modified as incision sites for the surgical procedures. In order to treat residual defects, the basic surgical strategy should be rearranging the scars to lie in the natural skin folds. This often necessitates a slight modification of standard flap designs. In addition, modification of the selected surgical procedures is needed to replace all missing elements of the face and simulate the original tissues as much as possible. From the esthetic point of view, this means the facial structures should simulate the original tissue and the adjacent tissues in terms of contour, thickness, color, texture, hair bearing, and skin elasticity and should be in balance with the adjacent tissues. Functionally, the reconstructed areas should also be able to participate in the normal facial movements, smiling, and speech. Such revisions and secondary operations are often necessary (1).

The ideal facial reconstruction should provide a good color and texture harmony with adjacent tissues. Minimal donor site necrosis and hidden scar are also desirable. Optimal esthetic reconstruction of facial defects is dependent on the availability of donor sites with color, texture, sebaceous quality, and thickness similar to the defective tissues. A great number of local flaps, pedicle flaps, and micro-vascular free flaps have been employed over the years for the reconstruction of mid-facial defects. Although facial trauma is a complex of hard tissue and soft tissue damage, we excluded combined damage and evaluated patient with only soft tissue damage to different parts of face. 

Forehead reconstruction is similar to the scalp; however, the esthetic considerations have greater importance. Conservative debridement and washing should be performed in the early stages. Local flaps based on supraorbital or supratrochlear vessels can be used in small defects. Tissue expansion is in priority in more serious defects due to the potential for color mismatches associated with skin grafting. As a result skin grafting is generally used to either allow transient wound closure until tissue expansion can be performed or to repair near complete forehead defects (21).

Most ear traumas are usually managed in the emergency room with the exception of subtotal or complete avulsion, which requires prompt surgical repair. Ear laceration must be repaired anatomically and initially requires helical rim realignment. Conservative debridement may be necessary for avoiding unnecessary cartilage exposure; albeit, potentially injured cartilage should be debrided because of the possible chondritis after closure. The perichondrium and skin can be closed in one layer using nonabsorbable suture. Mostly, skin grafting can be used in repairing of small skin defects with an intact perichondrium. In nonintact perichondrium, the resection of the underlying cartilage or a postauricular flap can be used for vascular supply. Replantation as a graft 12 hours after injury can be done in small avulsed segments. Staged reconstruction of the auricle has been done in larger avulsions or ear amputation; however, several authors have recommended dropping this technique because of repeated operations, cartilage resorption, and poor esthetic results. Recommendation for alternative method is either rib cartilage or the avulsed ear cartilage use as a mesh for later coverage and skin grafting (22, 23).

Reconstruction of eyelid lacerations has several key points. The lids have several layers and are very important for globe preservation; therefore, high accuracy is required for its reconstruction and each anatomic layer must be reconstructed separately. The eyelid or periocular injury is categorized to four classes according to the injured region (medial canthus, lateral canthus, upper eyelid, and lower eyelid) (24) and to two classes according to being a partial- or full-thickness injury. First, simple eyelid lacerations should be closed in three layers: conjunctiva, tarsus, and skin. Additionally, for lid margin lacerations, the gray line and tarsal plate must be carefully re-approximated to prevent notching. Proper alignment of lower lid lacerations minimizes the risk of ectropion in upper lid lacerations. The levator muscles should be carefully evaluated for damage of muscular insertions onto the tarsal plate. In injuries to medial aspect of the eye, lacrimal duct injuries should be ruled out.

Upper and lower eyelid full-thickness defects involving less than 33% and 50% of the eyelid length, can be closed primarily according to the principles described above (25). Some authors suggest primary closure only in upper-lid full- thickness defects involving up to 25% of the eyelid length (24). A lateral canthotomy and cantholysis can be used to decrease the tension of primary closure in larger defects. Local flaps, skin grafts, or regional flaps can be used in larger defects.

Partial-thickness defects of less than 50% of the eyelid length can, in contrast, be closed using local flaps. Partial-thickness defects involving greater than 50% of the upper or lower eyelid length typically require a full-thickness skin graft to achieve a tension-free closure. Full-thickness upper and lower eyelid defects that cannot be primarily closed require composite grafts or flaps. Near complete defects of the upper and lower eyelid are repaired using a switch flap, composite grafting, and cheek advancement flap. Repair of the injured canthus due to the injuries to the lateral eyelid commonly require either a canthopexy or canthoplasty. Primary repair, alternate repair, or recreation of the ligament might be required according to the severity of the injury. Medial canthal tendon and lacrimal system may be involved as a result of the injuries to the medial canthal region. Medial canthus injuries are commonly concomitant with fractures of the maxilla and nasal bone. Injuries to the lacrimal canaliculi can also occur in this region. Soft tissue defects can be closed using local flaps from the upper eyelid or glabella after repair of lacrimal system injury. Dorsalis pedis with septal cartilage, radial forearm flaps, and anterolateral thigh flaps can be used in complete eyelid and orbital reconstruction (26-28); however, complete prosthesis should be considered for significant injuries.

Nasal reconstruction is interesting for reconstructive surgeons and different reconstructive approaches to nasal trauma have been developed. Several algorithms have been made for the evaluation and reconstruction of nasal defects (29-31). If there is no skin defect in nasal laceration, primary repair is done. Anatomical lines alignment should be established exactly. Local flaps or full-thickness skin grafts are used to repair the skin defects. Reconstruction of the esthetic subunits of the nose has conflicting opinions. Commonly, if greater than 50% of a subunit is compromised, the remainder should be excised so that the entire subunit can be repaired monolithicaly (32). Firstly, the alar and tip must be repaired to prevent notching. If alar is involved, postauricular and supraclavicular full-thickness skin grafts can be used for partial-thickness defects over subunits with thinner skin; however, results are frequently suboptimal because of poor color match.

More commonly, local and regional flaps such as dorsal nasal, cheek advancement, nasolabial, and paramedian forehead flaps are used to repair soft tissue defects. Tissue expanders can also be used to expand the available tissues. Large severe traumatic defects and defects extending beyond the nose should be repaired with free flaps. Unfortunately, these patients have poor esthetic outcomes (33-37).

The cheeks are the largest subunit of the face and therefore, are exposed to multiple trauma. Many cheek wounds can be repaired primarily due to the laxity and availability of surrounding soft tissue. In small wounds, primary closure is often preferred. If not possible, local or regional flaps can be used to repair large defects due to the skin excess and laxity in the cheek. These flaps are generally preferred to skin grafting with regard to achieving soft tissue coverage. If none are possible, full-thickness skin grafting can be performed; however, the potential for scarring and contour deformities limit the use of this technique. Split-thickness skin grafts have potential for contractures that deform adjacent structures. Free flaps are also used for cheek reconstruction in more complex soft tissue defects. Local flaps for cheek reconstruction include Rhomberg transposition flap, advancement flap, rotation-advancement flap (Mustarde flap), and v-y advancement (kite flap) (38). Due to the unique characteristics of the cheek subunits, few complex local ﬂap designs can be used to reconstruct the vast majority of defects (38, 39).

Primary reconstruction is usually possible in lip lacerations. Correct alignment and a layered, tension-free closure of lip landmarks are important to proper lip injury management. The first suture should be done in vermilion and the exact alignment of the lips should be established. If the defect is up to 30%, it can be repaired primarily; however, larger defects require local flap for reconstruction taken from the opposite lip or cheek. The type of flap depends on the location and size of the defect. For defects of the central upper lip, primary closure may disrupt the normal anatomy of the philtral columns and dimple. For wounds that cannot be primarily closed, the best method to achieve restoration is to use available lip tissue for the repair. Although skin grafting can play an important role in management, color mismatches may be an issue when grafting vermillion defects. This can be corrected by later using an Abbe´ flap to reconstruct the central vermillion without moving the commissure. Larger defects or those involving other areas of the lip can be repaired using similar lip-switch procedures or a variety of local flaps (40-43).

The key element to a successful outcome is the usage of appropriate surgical technique. Prevention of complications like scars, infection, and long-term complication is very important. Analysis of the injury including location, size, and depth of penetration as well as presence of associated injuries will aid in the formulation of a proper surgical plan. As in all cases of plastic surgical planning, a strong understanding of the esthetic units of the face and the skin tension lines is important, especially when confronting a complex, irregular laceration. We recommend PDL therapy of suture lines early post operation (two weeks) after suture removal for better esthetic results. Revisions can always be performed later and after scar maturation.
